# Blood conservation strategies in cardiac valve replacement: A retrospective analysis of 1645 patients

**DOI:** 10.1097/MD.0000000000005160

**Published:** 2016-10-14

**Authors:** Junnan Zheng, Liangwei Chen, Linfeng Qian, Jianjie Jiang, Yinglian Chen, Jue Xie, Liping Shi, Yiming Ni, Haige Zhao

**Affiliations:** Department of Thoracic and Cardiovascular Surgery, The First Affiliated Hospital of Zhejiang University, School of Medicine, Hangzhou, China.

**Keywords:** blood conservation, cardiac valve replacement, cardiopulmonary bypass, intraoperative autologous donation, transfusion

## Abstract

We aimed to evaluate whether blood conservation strategies including intraoperative autologous donation (IAD) could reduce perioperative blood transfusion for patients undergoing cardiac valve replacement including mitral valve replacement, aortic valve replacement (AVR), and double valve replacement (DVR).

A total of 726 patients were studied over a 3-year period (2011–2013) after the implementation of IAD and were compared with 919 patients during the previous 36-month period (January 2008–December 2010). The method of small-volume retrograde autologous priming, strict blood transfusion standard together with IAD constituted a progressive blood-saving strategy.

Baseline characteristics and preoperative information showed no statistically significant difference between IAD group and non-IAD group. Most of the postoperative morbidities are statistically the same in the 2 groups. Chest tube output (415.2 vs 1029.8 mL, *P* < 0.001) and postoperative respiratory failure (5.9% vs 8.6%, *P* = 0.039) favored the IAD group, whereas hematocrit levels were more favorable in the non-IAD group (30.3% vs 33.0% at the end of the operation, *P* < 0.001; 30.4% vs 31.5% at the time of discharge). The use of blood product transfusion was higher in the non-IAD group (22.6% vs 43.3%, *P* < 0.001). Binary multivariate logistic regression analysis showed that high age, non-IAD, DVR surgery, and absent smoking history are associated with a higher risk of intra-/postoperative blood transfusion.

Blood conservation is effective and safe in cardiac valve replacement surgeries. The use of intraoperative autologous donation can lead to improved outcomes including a significantly lower rate of intra-/postoperative blood transfusion and postoperative complications.

## 1 Introduction

Blood transfusions are often required in patients who undergo heart surgery due to hemorrhage. Fifty percent of cardiac operations were reported to requiring blood transfusions in America^[1]^ compared with 70% in China.^[2]^ With the rapidly increasing number of surgical operations being performed in China, the limited level of blood donations cannot satisfy all of the patients’ requirements, and some hospitals even have to postpone surgery due to the lack of blood.^[3]^ Moreover, further studies have shown that allogenic blood transfusions can increase the incidence of infectious diseases, lung injury, and immunosuppression^[4,5]^ and also reduce short- and long-term survival.^[6,7]^

At present, it is well recognized that tolerance to permissive anemia (hemoglobin levels as low as 6 g/dL)^[1]^ during operations and cardiopulmonary bypass (CPB) is achievable and cost-effective, which provides a theoretical basis for the feasibility of preoperative autologous blood storage followed by intraoperative and postoperative autologous blood reinfusion. A recent study showed that more aggressive blood conservation strategies during CPB in cardiac surgery, such as intraoperative autologous donation (IAD), retrograde autologous priming (RAP), and shorter length circuit,^[8]^ could reduce the need for postoperative allogenic blood transfusions.

Rheumatic valvular disease is the most common reason for heart surgery in China.^[9]^ Valve replacement is an effective treatment that improves the quality of and prolongs the life of patients.^[10]^ Our previous study has demonstrated the advantages of IAD-based conservation strategies in mitral valve replacement (MVR) patients who were most commonly affected by rheumatoid causes.^[11]^ However, the role that IAD played in the whole valve placement population was still to be determined. To carry out a retrospective study, we collected data on patients who underwent an autologous transfusion from 2011 to 2013 and compared these with data from patients with no autologous transfusion from 2008 to 2010 to confirm the advantages of a blood conservation strategy based on IAD.

## 2 Materials and methods

**Figure 1:**
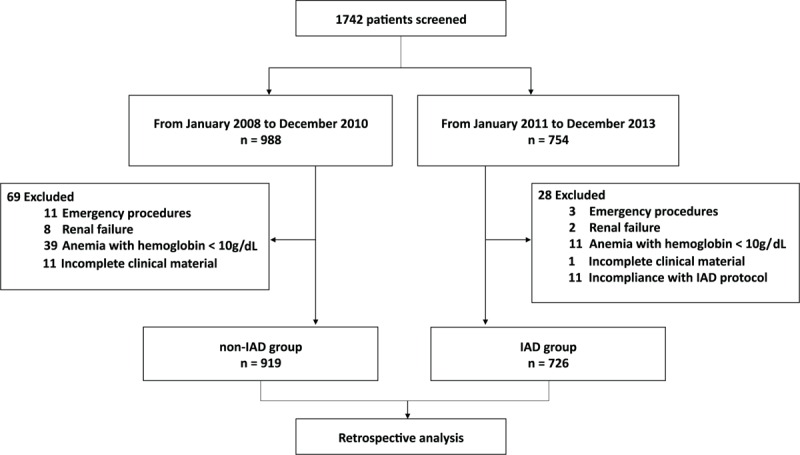
Consort flow diagram of patient enrollment.

After obtaining written informed consent waived by the Hospital Review Board, we retrospectively reviewed data from a database of a single surgical center, the Department of Cardiothoracic Surgery at the First Affiliated Hospital of Zhejiang University, China. The cohort included patients (aged 18 years and above) who underwent cardiac valve replacement surgeries using CPB during the 6-year period from January 2008 to December 2013. The 3 types of surgeries studied were MVR, aortic valve replacement (AVR), and double valve replacement (DVR). Exclusion criteria included emergency procedures, renal failure, infective endocarditis, preoperational hematocrit (HCT) <30% or hemoglobin <10 g/dL, patients with incomplete clinical material, and cases noncompliant with the IAD protocol (Fig. 1).

The 6-year period was divided equally into 2 3-year periods (non-IAD group and IAD group) before and after the implementation of IAD, which began at the end of 2010.

The autologous donation was performed before the initial systematic heparinization of cardiopulmonary bypass. Using a needle inserted into the purse string on the right auricle, the blood was siphoned into a blood bag (disposal plastic blood bag H31022624, Shanghai Transfusion Technology Co., Ltd.) for storage. Each blood bag contained 100 mL of blood preservation fluid (per 1000 mL: sodium citrate, 13.2 g; citric acid, 4.8 g; and glucose, 14.7 g) and was used to store 400 mL of blood. The blood bags were stored at room temperature in the operation room.

The IAD volume was based on the preoperational HCT level of the patients. For most of the patients (weight 50–70 kg), the IAD volume prescribed was 600 mL for patients with HCT > 40; 400 mL for patients with HCT 35 to 40, and 300 mL for patients with HCT 30 to 35. As for patients who weighed less than 50 kg, the IAD volume was decided as 400 mL for patients with HCT > 40, 300 mL for patients with HCT 35 to 40, and 200 mL for patients with HCT 30 to 35. However, for patients who weighed more than 70 kg, the IAD volume prescribed was 800 mL for patients with HCT > 40, 600 mL for patients with HCT 35 to 40, and 400 mL for patients with HCT 30 to 35.

During the IAD, the intravascular volume was replaced by an equal amount of crystalloid fluid to maintain euvolemia. The volume of IAD was stored and transfused after surgery when protamine administration had been completed.

During the operation, anticoagulation with 3 mg/kg of heparin was later neutralized by protamine (ratio 1:1) at the end of the CPB. Activated clotting time (480–600 seconds) was used to monitor anticoagulation, and no antifibrinolytics were used during CPB. Cardiotomy suction into the cardiopulmonary circuit reservoir was used. The Cell Saver blood salvage system (Cell Saver 5+, Haemonetics Corporation) and high performance hemoconcentrator (HPH 1400TS, HEMOCOR) were used during surgery to recover any blood that was shed and to concentrate the residual blood in the bypass circuit after surgery. Identical cardiopulmonary equipments were used in both groups, including stockert s3 heart lung machine (Sorin), affinity oxygenation system (Medtronic), and QUART arterial filter (Maquet). 4:1 cold blood cardioplegia was applied for both groups for myocardial protection.

In addition, except for the implementation of IAD, identical blood conservation strategies, including small-volume RAP and strict blood transfusion standards, were followed in both time periods. The volume of the circuit was approximately 1200 mL, and it was not heparin-coated. The volume of the circuit was identical during the 6-year period. The retrograde autologous priming volume, approximately 200 to 500 mL, was empirically collected for each patient in both groups to determine the efficacy, safety, and blood-saving effect of this specific volume.^[12]^

Perioperative blood transfusions were performed using a validated transfusion protocol.^[13,14]^ The triggers for intraoperative transfusion of packed red blood cells (PRBCs) included hemoglobin levels <6 g/dL or HCT level <18% and, in addition, at least one of the following during CPB: partial pressure of oxygen <60 mm Hg, lactate level >2.2 mmol/L, base deficit >3 mEq/L, and serum bicarbonate level <22 mEq/L. Triggers for postoperative PRBC transfusion included hemoglobin levels <7 g/dL or HCT level <21% and, in addition, at least one of the following: elevated oxygen requirement (fraction of inspired oxygen ≥60), persistent systemic hypotension (systolic blood pressure <90 mm Hg) despite the use of vasopressors, evidence of end-organ dysfunction, or evidence of persistent bleeding (>200 mL/h for at least 3 hours). Triggers for platelet transfusion included platelet count had reached 10,000 μ/L or less, platelet count had reached 50,000 μ/L or less in addition to bleeding >1.5 mL/kg/h for 2 consecutive hours, and uncontrolled profuse microvascular bleeding after surgery. Triggers for the transfusion of fresh frozen plasma included unmanageable hemorrhage after protamine neutralization, disseminate intravascular coagulation, preoperatively confirmed coagulation factor deficiency, emergency reversion of the oral anticoagulant effect, and duration of CPB more than 2 hours.

Using SPSS software for windows (version 22.0), the data were analyzed and presented as means, numbers, or ratios as needed. Gaussian distributed data were reported as mean ± standard deviation. Non-Gaussian distributed data were reported as median (interquartile range). The null hypothesis set was that patient outcomes would not differ significantly. Continuous variables were expressed as means depending on overall variable distribution, and categorical variables were expressed as standard group percentages. Pearson χ^2^ test was used for descriptive, univariate statistics, while the *t* test was used for normally distributed data comparisons. The Mann–Whitney *U* test was used for group comparison of continuous non-Gaussian distributed variables. Two-tailed *P* values were derived from the calculated test statistics. A multivariate logistic regression model was performed to determine the risk factors for intra-/postoperative blood transfusions.

## 3 Results

A total of 988 (60.2%) patients underwent cardiac valve replacement surgery from January 2008 to December 2010 compared with 754 (39.8%) during the 3-year period after the implementation of IAD (from 2011 to 2013). After exclusions, 919 (55.9%) patients entered our study as the non-IAD group, and 726 (44.1%) patients were included in the IAD group.

Table 1 describes the baseline characteristics, demographics, and comorbidities for the patients in both groups.

**Table 1:**
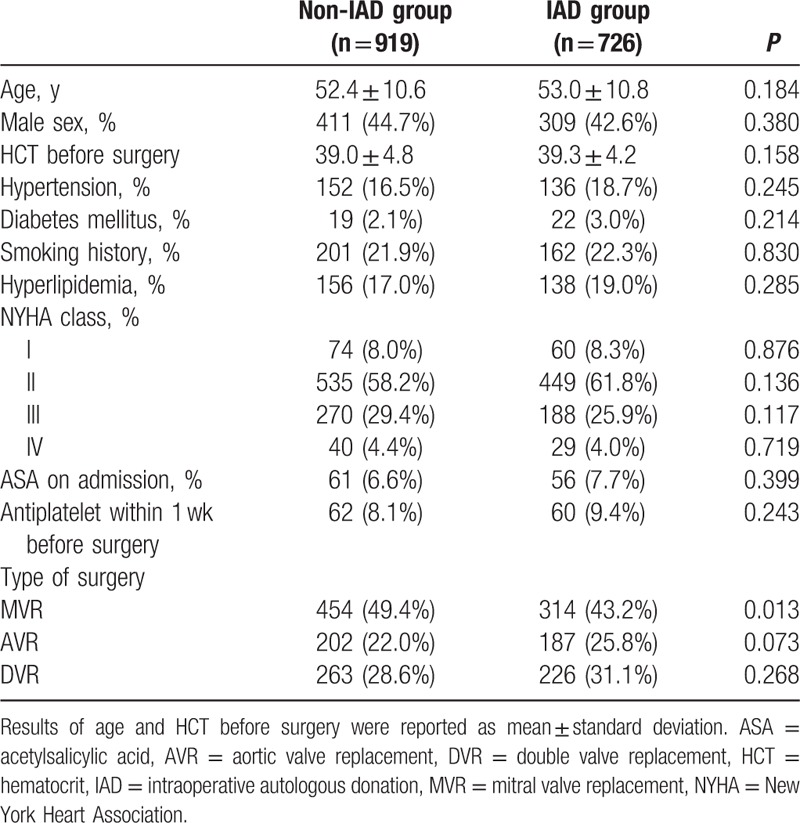
Patient baseline information.

As can be seen in Table 1, there were no significant differences regarding most of the baseline characteristics. No obvious difference in the central blood volume or incidence of severe pulmonary hypertension was seen between the 2 groups (data not shown). The types of surgeries performed were mostly equivalent in both groups with the exception of MVR (49.4% vs 43.2%, *P* = 0.017).

Table 2 summarizes the CPB parameters, intraoperative characteristics, and postoperative outcome of the patients. The duration of CPB time, the rates of various postoperative morbidity, length of stay in hospital, and death within 30 days were statistically similar in the 2 groups. However, chest tube output and postoperative respiratory failure favored the IAD group. As can be seen in Table 2, the use of blood product transfusion was higher in the non-IAD group. Fewer IAD patients received intra-/postoperative transfusions of blood products. However, HCT levels were more favorable in the non-IAD group (at the end of the operation and at the time of discharge).

**Table 2:**
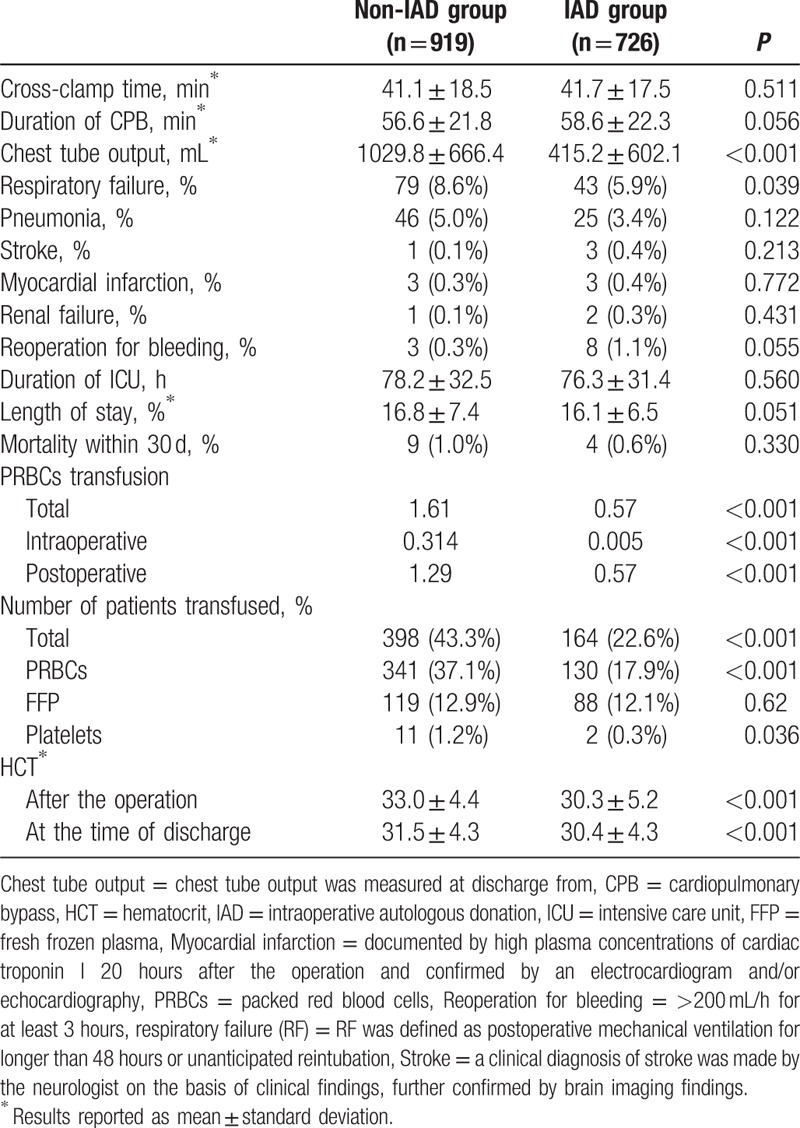
CPB parameters, intraoperative characteristics, and outcomes of the patients.

According to the baseline characteristics of the 2 groups (Table 1), a multivariate logistic regression model was constructed to determine the risk factors for blood transfusions and to predict the probability of the use of PRBCs (Table 3). In the logistic model, the IAD group received significantly fewer PRBC transfusions (*P* < 0.001). Older age and DVR surgery were associated with an increased need for blood transfusions. In addition, patients with history of smoking were less likely to use allogenic blood products. Further, we compared HCT levels between smokers and non-smokers and found out that smokers had significantly higher HCT levels than nonsmokers (HCT before surgery 40.31 vs 38.84, *P* < 0.001; HCT after surgery 33.57 vs 31.32, *P* < 0.001; and HCT at the time of discharge 31.77 vs 30.8, *P* < 0.001). Our multivariate logistic model is based on baseline characteristics on admission; therefore, HCT levels were not included in the analysis of risk factors.

**Table 3:**
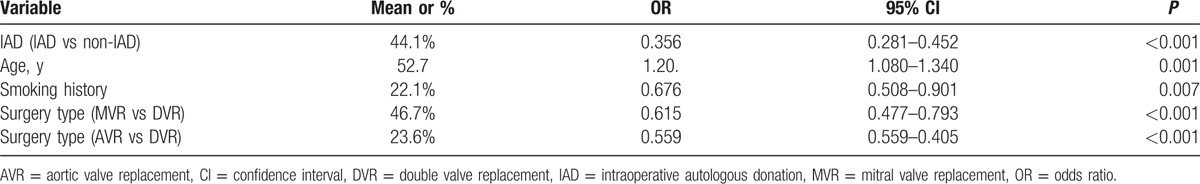
Multivariate logistic regression model for allogenic packed red blood cell transfusion.

Using the former multivariate logistic regression model, the following formula was devised to calculate the probability of intra-/postoperative PRBC transfusions for cardiac valve replacement patients: 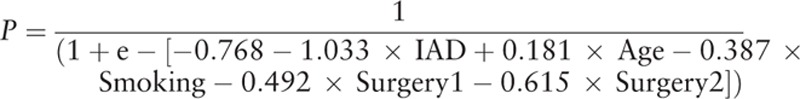


IAD: 1 for patients who underwent IAD and 0 for patients who did not; Age: years; Smoking: 1 for patients with a history of smoking and 0 for patients who did not; Surgery: Surgery1 = 1 with Surgery2 = 0 for MVR, Surgery1 = 0 with Surgery2 = 1 for AVR, and Surgery1 = 0 with Surgery2 = 0 for DVR patients.

## 4 Discussion and conclusion

Patients who undergo cardiac surgery use nearly 20% of the total blood bank in the United States.^[15]^ Traditionally, avoiding anemia can improve the prognosis of patients,^[16]^ but further evidence has proved that perioperative allogenic blood transfusions during cardiac surgery can be related to higher morbidity, mortality, and hospitalization costs.^[17]^

We carried out autologous blood transfusions to achieve a maximum reduction of allogenic blood transfusion, in accordance with 2011 American College of Surgeons and The Society of Thoracic Surgeons guidelines.^[1]^ Our method is not complicated, but requires the cooperation of surgeons, cardiologists, anesthetists, percussionists, intensivists, and nursing staff.

Our results showed a significant decrease in allogenic blood transfusions in the group who underwent the autologous blood transfusion (0.57 vs 1.61 units, *P* < 0.001) and significantly fewer IAD patients received intra-/postoperative transfusions of blood products (22.6% vs 43.3%, *P* < 0.001). Our result also showed that postoperative chest tube output favored the IAD group (415.2 vs 1029.8 mL, *P* < 0.001), similar results had been reported in literature.^[8]^ The specific mechanism of it has not been established; however, we assumed that there are several explanations. First, patients in non-IAD group had more intraoperative transfusion of banked blood, which might be associated with a poorer coagulation function. Also, in the IAD group, a certain amount of self-donating blood is siphoned into a blood storage bag before the initiation of systematic heparinization and transfused back after surgery, skipping the process of cardiopulmonary bypass. Therefore, to some extent, the platelet function of this amount of blood can be preserved. On the other hand, decreased transfusions are known to result in fewer respiratory complications, which was also confirmed in our study (5.9% vs 8.6%, *P* = 0.039). However, no significant differences were found regarding myocardial infarction, pneumonia, or renal failure, which was consistent with other studies.^[13]^

Unexpectedly, postoperative and at-discharge levels of HCT of the patients in the autologous blood transfusion group were lower than those of the non-IAD group. Hajjar et al^[18]^ found no significant difference in the mortality and complication rates between 2 groups of patients undergoing cardiac surgery with perioperative HCT levels of 30% and 24%, respectively. In our study, although patients in IAD group showed lower HCT levels at the end of the operation (30.0 vs 33.3) and at the time of discharge (30.4 vs 31.5), such relatively low levels would not and did not affect the survival rate of patients.

We found that patients with a history of smoking had fewer transfusions of allogenic PRBCs, intraoperatively and postoperatively, than those with no smoking history, and fewer blood transfusions in smokers after heart procedures have been reported in other studies.^[19,20]^ We compared HCT levels and found out that smokers had significantly higher HCT levels than nonsmokers. The combination of carbon monoxide in tobacco and the effects of nicotine disrupts oxygen delivery to the tissues and stimulates the bone marrow to produce more red blood cells and thereby increase HCT and hemoglobin.^[21]^ Thus, in this study, high HCT levels in smokers were associated with fewer PRBC transfusions.

Our results indicated that older patients have higher risk of needing allogenic blood transfusions. Age has been reported to be one of the independent risk factors for perioperative bleeding in many studies.^[22]^ Balleisen et al^[23]^ believed that due to the high blood viscosity and slow blood flow in elderly patients, blood is hypercoagulable. The CPB process accelerates coagulation, entailing large amounts of blood clotting factors, which leads to a decrease in blood coagulation and thus an increase in the risk of bleeding.

These results should be interpreted in the light of several limitations. As a retrospective analysis, the study design had some inherent disadvantages. The implementation of the IAD-based blood conservation strategy was introduced after the patients in the non-IAD group had been treated; thus, slight changes or improvements in clinical care or discharge criteria might have occurred over time and influenced such indices as duration of hospital stay and other factors. Also, although strict and identical intra-/postoperative transfusion standards were introduced in both periods, potential incompliance with the transfusion protocol might occur at the beginning of the implementation of IAD, thus might explain the difference of postoperative HCT between the 2 groups. Another limitation is that the recorded differences in patient outcomes could originate from smaller recorded or unrecorded differences between the 2 groups. Due to the disadvantage of the retrospective analysis, several characteristics and medical indices of the patients, including central blood volume, chest tube output in terms of different time periods, and others, were omitted. Therefore, a randomized clinical trial is needed for further study.

In conclusion, our study showed that IAD-based blood conservation strategies can be effective and safe in cardiac valve replacement surgery. A certain degree of intraoperative and postoperative anemia would not affect the prognosis of the patients, and patients who adopted the autologous blood transfusion had better outcomes with regard to blood transfusion, postoperative chest tube output, and respiratory dysfunction. We therefore urge that more large-scale multicenter randomized clinical trials be carried out worldwide to prove the efficacy and safety of intraoperative autologous blood transfusions in cardiac surgery.

## Acknowledgments

The authors thank the anesthetists, the staff of the CPB division, and laboratory department in the First Affiliated Hospital of Zhejiang University for collection and managing the data presented in this report.
